# Evaluation of factors affecting Pneumonia Severity Index and antibiotic resistance status in culture-proven bacterial pneumonia

**DOI:** 10.3389/fmed.2026.1720340

**Published:** 2026-02-03

**Authors:** İlknur Kaya, Aynur Gülcan, İnci Arıkan

**Affiliations:** 1 Department of Chest Diseases, Faculty of Medicine, Kütahya Health Sciences University, Kütahya, Türkiye; 2 Department of Medical Microbiology, Faculty of Medicine, Kütahya Health Sciences University, Kütahya, Türkiye; 3 Department of Public Health, Faculty of Medicine, Kütahya Health Sciences University, Kütahya, Türkiye

**Keywords:** culture-proven bacterial pneumonia, multidrug resistance, pneumonia, Pneumonia Severity Index (PSI), pneumonia pathogens

## Abstract

**Introduction:**

The Pneumonia Severity Index (PSI) is a widely used to assess disease severity and guide hospitalization decisions in patients with pneumonia. However, PSI does not incorporate microbiological characteristics, antimicrobial resistance patterns, or immune–inflammatory biomarkers. This study aimed to evaluate the impact of comorbidities, etiological agents, antimicrobial resistance, and immune–inflammatory markers on PSI scores in patients with culture-proven bacterial pneumonia.

**Materials and methods:**

This retrospective study included patients with culture-proven bacterial pneumonia treated at a tertiary care center between January 2019 and April 2023. Demographic characteristics, comorbidities, intensive care unit (ICU) admission, etiological agents, antimicrobial resistance patterns, and multidrug resistance (MDR) status were analyzed. Laboratory parameters, including neutrophil-to-lymphocyte ratio (NLR), platelet-to-lymphocyte ratio (PLR), monocyte-to-lymphocyte ratio (MLR), systemic immune–inflammation index (SII), C-reactive protein, and albumin levels were evaluated in relation to PSI scores.

**Results:**

Most patients were aged ≥65 years and had at least one comorbidity. Gram-negative bacteria predominated, with *Pseudomonas aeruginosa* being the most frequently isolated pathogen. Multidrug resistance was most commonly observed in *Acinetobacter baumannii*, *Klebsiella* spp., and *Escherichia coli*. Higher PSI scores were significantly associated with a greater comorbidity burden, presence of MDR pathogens, elevated NLR, PLR, MLR, and SII values, and lower albumin levels (*p* < 0.05). Patients with MDR isolated had significantly higher mean PSI scores than those without MDR.

**Conclusion:**

In culture-proven bacterial pneumonia, comorbidity burden, antimicrobial resistance–particularly multidrug resistance–and elevated immune–inflammatory biomarkers are associated with higher PSI scores. These findings highlight the importance of considering community-onset healthcare-associated pneumonia and integrating local antimicrobial resistance surveillance and laboratory biomarkers into clinical risk stratification. Further multicenter prospective studies are warranted to validate these associations.

## 1 Introduction

Community-associated pneumonia (CAP) is a common and potentially life-threatening infection associated with substantial morbidity and mortality worldwide and is defined as a pneumonia occurring in individuals without recent contact with the healthcare system (1). Health care-associated pneumonia (HCAP) is no longer considered as a separate entity but as a form of CAP and is also defined as pneumonia present at the time of hospital admission or developing within 48 h thereafter in patients with recent healthcare exposure, including hospitalization, dialysis, or residence in long-term care facilities or coexists with a family member with multi-drug resistant pathogen (2–4).

Despite advances in vaccination strategies and antimicrobial therapy, pneumonia-related mortality has remained largely unchanged over recent decades (5). The etiological spectrum of pneumonia and antimicrobial resistance patterns varies according to geographic region, patient characteristics, and healthcare exposure. Increasing attention has therefore been directed toward Gram-negative pathogens and antimicrobial resistance, particularly in patients with multiple comorbidities (1, 2).

Identification of the causative pathogen in hospitalized pneumonia remains challenging, with culture positivity rates reported to be limited even in large surveillance studies (6, 7). Consequently, clinical severity assessment tools such as the Pneumonia Severity Index (PSI) are widely used to guide management decisions and estimate prognosis (8). However, PSI primarily incorporates demographic and clinical variables and does not directly account for microbiological characteristics, antimicrobial resistance, or immune–inflammatory biomarkers.

Although the prognostic value of inflammatory markers such as neutrophil-to-lymphocyte ratio (NLR), platelet-to-lymphocyte ratio (PLR), and systemic immune–inflammation index (SII) have been investigated in various infectious diseases, including pneumonia, their relationship with pathogen profile, multidrug resistance, and disease severity in culture-proven bacterial pneumonia remains incompletely defined, particularly in populations with a high comorbidity burden.

The aim of this study was to evaluate factors influencing PSI scores in patients with culture-proven bacterial pneumonia, with particular focus on comorbidities, etiological agents, antimicrobial resistance patterns, and selected immune–inflammatory laboratory parameters.

## 2 Materials and methods

This retrospective study was conducted in the Chest Diseases Clinic of Kütahya Health Sciences University Faculty of Medicine, Evliya Çelebi Training and Research Hospital. Ethical approval was obtained from the Non-Interventional Clinical Research Ethics Committee of the Faculty of Medicine, Kütahya Health Sciences University (Decision No. 2023/13-19, dated November 28, 2023).

Between January 1, 2019 and April 30, 2023, all patients (≥18 years) who were admitted to the chest diseases outpatient clinic, hospitalized in the chest diseases ward or intensive care unit of KHSU Evliya Çelebi Training and Research Hospital, and diagnosed with pneumonia based on clinical and radiological findings were included in the study if they had positive sputum, bronchial lavage, or tracheal aspirate cultures. Posteroanterior chest radiography (PAAG) and computed thoracic tomography (CT) data were used for radiological evaluation, which was performed by chest physicians.

Community-onset healthcare-associated pneumonia was defined as pneumonia present at hospital admission or developing within 48 h in patients with recent healthcare exposure, including hospitalization, dialysis, or residence in long-term care facilities. Due to the high prevalence of comorbid conditions and prior healthcare exposure a substantial proportion of patients met the criteria for community-onset healthcare-associated pneumonia.

Comorbidities were categorized as immunosuppressive (IS) conditions, chronic lung disease, or the coexistence of both. Immunosuppressive conditions included diabetes mellitus (DM), chronic renal failure (CRF), organ transplantation, malignancy other than lung cancer, ulcerative colitis, autoimmune disease (AID), and acquired immunodeficiency disease. Chronic lung diseases included chronic obstructive pulmonary disease (COPD), asthma, chronic restrictive lung disease (CRLD), bronchiectasis, cystic fibrosis, lung cancer, pulmonary embolism, and concomitant COVID-19 infection.

The Pneumonia Severity Index (PSI) was calculated by a pulmonologist based on demographic variables (age, sex, and nursing home residence); comorbidities (congestive heart failure, cerebrovascular disease, malignancy, and renal and liver disease); physical examination findings (tachypnea, tachycardia, blood pressure, confusion, and fever); and laboratory findings (arterial pH, urea, sodium, glucose, hematocrit, and arterial oxygen pressure). Patients with PSI scores of 0–90 were classified as PSI classes I–III, whereas those with scores of 91–130 and >130 were classified as PSI classes IV and V, respectively.

Lower respiratory tract samples sent to the microbiology laboratory were inoculated onto blood agar, eosin methylene blue (EMB) agar, and chocolate agar supplemented with bacitracin, and incubated at 37 °C for 24–48 h. Bacterial identification and antimicrobial susceptibility testing were performed using conventional methods or an automated system (BD Phoenix, USA). Antibiotics used for susceptibility testing and interpretation of results were applied in accordance with EUCAST version 15. Bacteria resistant to at least one antimicrobial agent in three or more antimicrobial categories were defined as multidrug-resistant (MDR). Microbiological data were grouped according to the causative microorganism. In the analysis of causative agents and antimicrobial resistance, only the first isolate was included when the same bacterium was consecutively isolated from the same type of clinical samples obtained from the same patient, in accordance with cumulative antibiogram analysis guidelines (CLSI M39-A4).

All data, including sociodemographic characteristics (age, sex, outpatient or inpatient status), clinical data (comorbidities and PSI), and laboratory data (culture results, C-reactive protein, complete blood count, and albumin), were entered into the IBM SPSS Statistics version 25.0 software using anonymized data collection forms.

### 2.1 Statistical analysis

Study data were analyzed using IBM SPSS Statistics version 25.0. Frequency distributions and percentages were calculated for descriptive analyses. Continuous variables were summarized as mean ± standard deviation or median (minimum–maximum), depending on their distributional characteristics. The normality of continuous variables was assessed using the Kolmogorov–Smirnov and Shapiro–Wilk tests, as well as visual inspection of histograms and Q–Q plots. As none of the continuous variables showed a normal distribution, non-parametric methods were applied throughout the analysis.

For group comparisons, PSI scores were categorized as PSI classes I–III and PSI classes IV–V according to established clinical cut-off values indicating low and high pneumonia severity, respectively. The Mann–Whitney U test was used to compare continuous variables between two groups, whereas comparisons among more than two groups were performed using the Kruskal–Wallis test. When the Kruskal–Wallis test indicated statistical significance, Bonferroni-corrected *post hoc* pairwise analyses were conducted.

Categorical variables were analyzed using the chi-square test or Fisher’s exact test, as appropriate, based on expected cell counts.

The association between PSI score and inflammatory markers– NLR, PLR, MLR, SII, CRP, and albumin–was examined using Spearman’s rank correlation analysis due to the non-normal distribution of these variables. Although multivariable regression analysis could provide additional insight, the limited sample size within subgroups and the heterogeneity of comorbid conditions increased the risk of model overfitting and unstable estimates. Therefore, multivariable analysis was not performed.

A *p*-value of <0.05 was considered statistically significant for all analyses.

## 3 Findings

### 3.1 Demographic data

**FIGURE 1:**
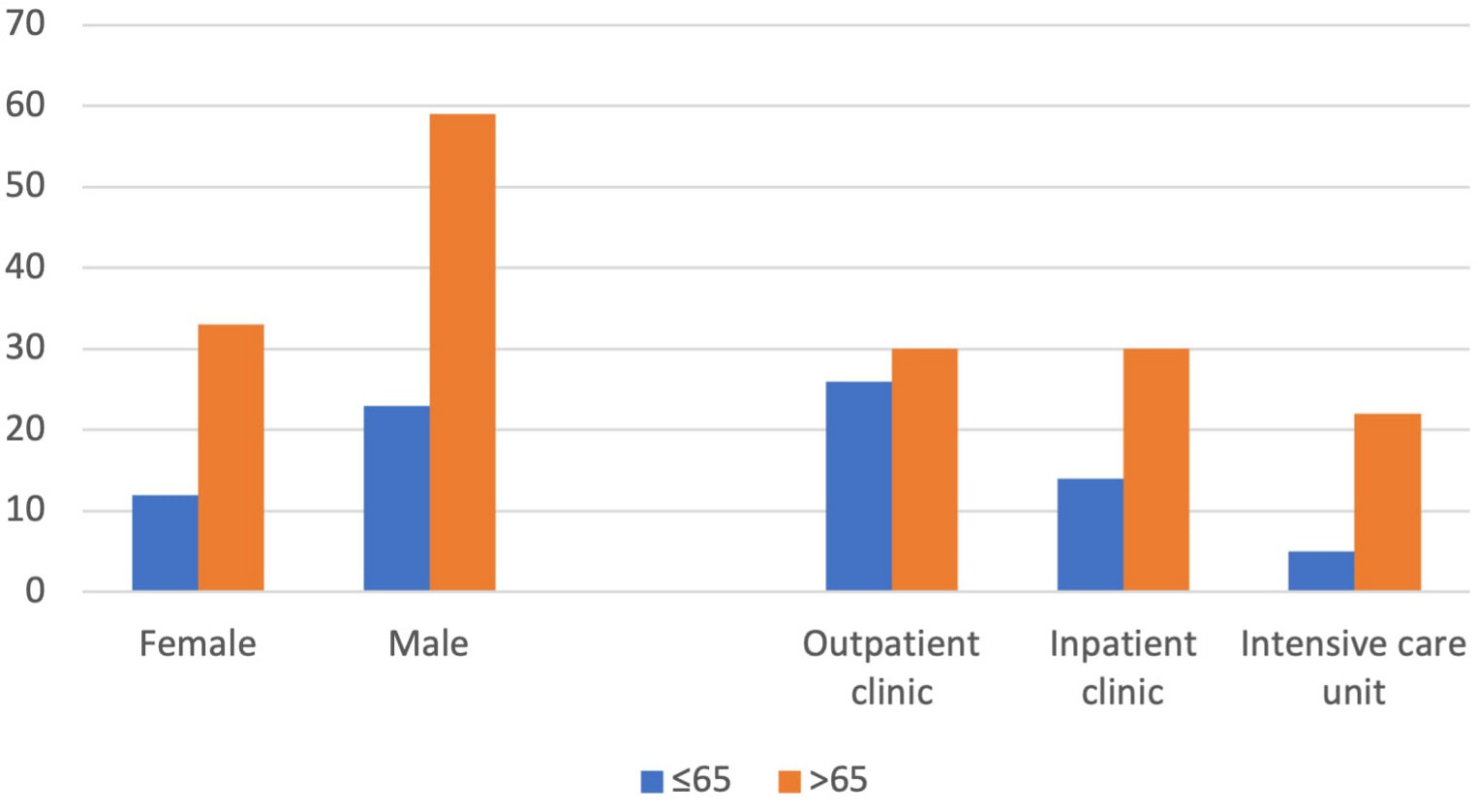
Distribution of gender by age and the units in which the patients were followed up.

Between January 1, 2019 and April 30, 2023, a total of 151 episodes of culture-proven pneumonia occurred in 127 patients. Of these patients, 64.6% were aged ≥65 years. Ninety-two patients (72.4%) were male (mean age ± SD: 68.6 ± 12.4 years), and 35 (27.6%) were female (mean age ± SD: 65.9 ± 17.1 years). Of the 151 pneumonia episodes, 71 (47.0%) were managed in the outpatient setting, 46 (30.5%) in the general ward, and 34 (22.5%) in the intensive care unit. The distribution of patients according to age, sex, and treatment setting is presented in Figure 1.

### 3.2 Investigation of factors affecting the pneumonia severity index

**FIGURE 2:**
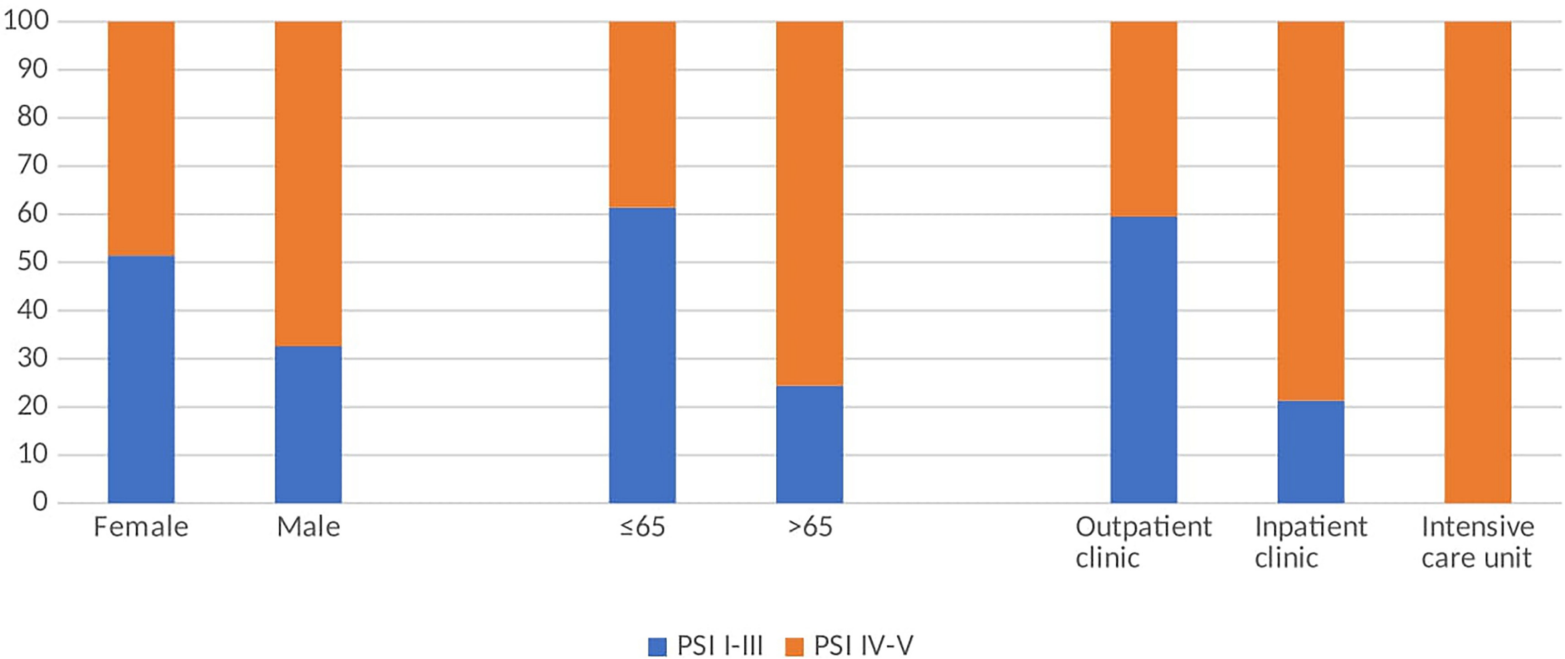
Percentages of low and high PSI according to gender, age and unit.

Among the 127 patients, 48 were categorized as PSI classes I–III and 79 as PSI classes IV–V. Higher pneumonia severity was significantly associated with age ≥65 years, male sex, and admission to the intensive care unit (*p* < 0.001, *p* = 0.05, and *p* < 0.001, respectively; Figure 2).

The majority of patient had an underlying lung disease and/or immunosuppressive (IS) conditions. We investigated the effect of lung disease alone, IS alone, and their coexistence on PSI. Although the proportion of patients with high PSI was 61.5% in those with lung disease alone and 65.5% in those with concomitant IS status, there was no statistically significant difference between them (*p* = 0.844) (Table 1). A detailed comparison of individual comorbidities according to PSI category is presented in Table 2. The most common comorbidities were airway diseases, including asthma and COPD (n:66) and DM (n:30), and patients with these comorbidities mostly had high PSI (Table 2).

**TABLE 1:** Association between patient comorbidities and Pneumonia Severity Index (PSI).

Comorbidity	PSI I–III	PSI IV–V	*P*-value
	*n* (%)	*n* (%)	
Lung disease only (n:52)	20 (38.5)	32 (61.5)	0.844
IS status only (n:25)	8 (32)	17 (68)	
Lung disease + IS status (n:29)	10 (34.5)	19 (65.5)	
**Number of comorbidities**			
<3 (n:79)	36 (45.6)	43 (54.4)	0.020
≥3 (n:48)	12 (25)	36 (75)	

IS, immunosuppressive. Statistical analysis was performed using Pearson’s Chi-square test. A *p*-value < 0.05 was considered statistically significant.

**TABLE 2:** Distribution of patient comorbidies according to the PSI classes.

Patient comorbidities	PSI I–III	PSI IV–V
Immunosuppressive conditions	*n* (%)	*n* (%)
None (n:77)	31 (40.3)	46 (59.7)
DM (n:26)	10 (38.5)	16 (61.5)
CRF (n:3)	0	3 (100)
Organ transplant (n:1)	1 (100)	0
Cancer (n:12)	1 (8.3)	11 (91.7)
Ulcerative colitis (n:1)	1 (100)	0
Autoimmune disease (n:1)	1 (100)	0
DM + Autoimmune disease (n:1)	1 (100)	0
DM + cancer (n:3)	0	3 (100)
Autoimmune disease + cancer (n:1)	1 (100)	0
Acquired immunodeficiency (n:1)	1 (100)	0
**Lung disease**		
None (n:43)	17 (39.5)	26 (60.5)
Asthma-COPD (n:63)	23 (36.5)	40 (63.5)
CRLD (n:1)	0	1 (100)
Bronchiectasis (n:7)	5 (71.4)	2 (28.6)
Cystic fibrosis (n:1)	1 (100)	0
Lung cancer (n:6)	0	6 (100)
Pulmonary embolism (n:1)	0	1 (100)
COPD + Lung cancer (n:3)	0	3 (100)
COVID-19* (n:2)	2 (100)	0

COPD, chronic obstructive pulmonary disease; CRLD, chronic restrictive lung disease; DM, diabetes mellitus; CRF, chronic renal failure; COVID-19, Coronavirus disease-2019. Statistical analysis was performed using Pearson’s Chi-square or Fisher’s Exact test. A *p*-value < 0.05 was considered statistically significant. *There were a total of 8 patients with post-COVID-19 lung disease in the study, and six of them were accompanied by pulmonary and extrapulmonary comorbidities. In the table, cases with post-COVID-19 lung disease without other comorbidities are indicated.

In addition, when effect of comorbidity number was evaluated, PSI was higher in patients with ≥3 comorbidities than in those with <3 comorbidities, there was a statistically significant difference (*p* = 0.020) (Table 1).

### 3.3 Distribution of isolated bacteria and multidrug resistance rates

A total of 165 bacteria were isolated from 151 episodes in 127 patients. The isolated agents in outpatients were *Pseudomonas aeruginosa* (32.4%), *Klebsiella* spp. (14.9%), *E. coli* (10.8%) and *Streptococcus pneumoniae* (10. 8%), while *P. aeruginosa* (26.1%), *Klebsiella* spp. (21.7%), *Acinebocter baumannii* (19.6%) and *Staphylococcus aureus* (10.9%) were found in ward patients. In intensive care unit patients, *Klebsiella* spp. (38.9%), *A. baumannii* (27.8%), *P. aeruginosa* (25%) and *S. aureus* (5.6%) were the most common organisms. The rate of underlying lung disease in patients from whom *P. aeruginosa* was isolated was 75% and 85.7% (p:0.076) in outpatients and inpatients, respectively, while the presence of immunosuppressive status was 52.9% and 37.5% (p:0.628).

Multidrug resistance was most frequently detected in *A. baumannii* (84.2%), *Klebsiella* spp. (77.4%) and *E. coli* (66.7%). This rate was much lower for *P. aeruginosa*. Multidrug resistance and resistance rates of the isolated bacteria to the most frequently used antibiotic categories in empirical treatment are given in Table 3.

**TABLE 3:** Rates of multidrug resistance (MDR) and empirical antibiotic resistance of pneumonia pathogens.

Bacteria *(Genus/species)*	MDR (+) (%)	3.SS CRO/CTX	AP.SS CAZ/FEP	CP IPM/MEM	QN LEV	P	BL-BLİ AMC/TZP	Mk
*P. aeruginosa*	32.5	-*	35.9/30.8	28.2/20	30	–	-/27.5	–
*Klebsiella* spp.	77.4	73.3	73.3/63.3	60/60	76.7	–	89.7/0	–
*Acinetobacter* spp.	84.2	-*	0/0	94.7/94.7	94.7	–	–	–
*E. coli*	66.7	75	45.5/50	8.3/8.3	66.7	–	60/33.3	–
*S. aureus*	0	–	–	–	20	100	9.1	27.3
*S. pneumoniae*	20	0	–	–	0	10	–	20
*H. influenza*	50	0	–	–	66.7	–	66.7	–
*Enterobacter* spp.	33.3	0	33.3/16.7	0/0	16.7	–	-*	–
*M. catarrhalis*	0	0	–	–	0	–	0	0
*Serratia* spp.	0	0	0/0	0	33.3	–	-*	–

3.SS, 3rd generation cephalosporin; AP.SS, antipseudomonal cephalosporin; CP, carbapenem; QN, quinolone; P, penicillin; BL-BLI, beta lactam-beta lactamase inhibitor; Mk, macrolide; CRO, ceftriaxone; CTX, cefotaxime; CAZ, ceftazisdim; FEP, cefepime; IPM, imipenem; MEM, imipenem meropenem; LEV, levofloxacin; AMC, amoxicillin clavulanate; TZP, piperacillin tazobactam. Statistical analysis was performed using Pearson’s Chi-square or Fisher’s Exact test. A *p*-value < 0.05 was considered statistically significant. *Naturally resistant.

A total of 165 bacterial isolates were obtained from 151 pneumonia episodes in 127 patients. Among outpatients, the most frequently isolated pathogens were *Pseudomonas aeruginosa* (32.4%), *Klebsiella* spp. (14.9%), *Escherichia coli* (10.8%), and *Streptococcus pneumoniae* (10.8%). In patients hospitalized in the general ward, *P. aeruginosa* (26.1%), *Klebsiella* spp. (21.7%), *Acinetobacter baumannii* (19.6%), and *Staphylococcus aureus* (10.9%) were the predominant pathogens. Among intensive care unit patients, *Klebsiella* spp. (38.9%), *A. baumannii* (27.8%), *P. aeruginosa* (25.0%), and *S. aureus* (5.6%) were the most commonly isolated organisms.

Among patients from whom *P. aeruginosa* was isolated, the prevalence of underlying lung disease was 75.0% in outpatients and 85.7% in inpatients (*p* = 0.076), whereas the prevalence of immunosuppressive status was 52.9% and 37.5%, respectively (*p* = 0.628).

Multidrug resistance was most frequently observed in *A. baumannii* (84.2%), *Klebsiella* spp. (77.4%), and *E. coli* (66.7%), whereas the rate of multidrug resistance was considerably lower in *P. aeruginosa*. Detailed data on multidrug resistance and resistance rates of the isolated bacteria to the most commonly used antibiotic classes in empirical therapy are presented in Table 3.

### 3.4 Comparison of laboratory data and PSI

Higher PSI scores on admission were significantly associated with older age, male sex, and increased likelihood of intensive care unit admission. Patients with clinical samples positive for *Klebsiella* spp. (85.7%), *A. baumannii* (85.7%), *H. influenzae* (80%), *E. coli* (66.7%), *S. aureus* (63.6%) and *P. aeruginosa* (59.1%), had predominantly high PSI, while patients with *Serratia marcescens* (25%), *M. catarrhalis* (20%) and *S. pneumoniae* (20%) had predominantly low PSI.

The mean PSI score (±SD) was significantly higher in patients with multidrug resistant isolates compared with those without multidrug resistance (133.09 ± 46.49 vs. 100.1 ± 43.55, *p* < 0.001). The comparison of PSI according to the isolated pathogens is presented in Table 4.

**TABLE 4:** Distribution of causative agents according to the PSI classes.

Causative microorganism	PSI I–III	PSI IV–V	*P*-value
	*n*%	*n*%	
*Klebsiella* spp. (n:35)	5 14.3	30 85.7	< 0.001
*E. coli* (n:12)	4 33.3	8 66.7	
*Enterobacter* spp. (n:6)	4 66.7	2 33.3	
*Serratia marcescens* (n:4)	3 75.0	1 25.0	
*Pseudomonas aeruginosa* (n:44)	18 40.9	26 59.1	
*Acinetobacter baumannii* (n:21)	3 14.3	18 85.7	
*Haemophilus influenza* (n:5)	1 20.0	4 80.0	
*Moraxella catrarrhalis* (n:4)	3 75.0	1 25.0	
*Streptococcus pneumoniae* (n:10)	8 80.0	2 20.0	
*Staphylococcus aureus* (n:11)	4 36.4	7 63.6	
**Multiple drug resistance**			
Absent	41 50.6	40 49.4	< 0.001
Present	13 16.9	64 83.1	

Statistical analysis was performed using Pearson’s Chi-square or Fisher’s Exact test. A *p*-value < 0.05 was considered statistically significant.

When evaluated according to immune-inflammatory parameters, NLR, PLR, MLR, SII, CRP values were significantly higher in patients with high PSI, whereas albumin values were significantly lower (Table 5).

**TABLE 5:** Median (IQR) values of immune-inflammatory parameters according to PSI classes.

Immune-inflammatory parameters	PSI I–III	PSI IV–V	
	Median (IQR)	(Median) (IQR)	*P*-value
NLR	3.081 (1.89)	7.305 (4.59)	< 0.001
PLR	147.3 (111.4)	193.87 (114.5)	0.014
MLR	0.31 (0.19)	0.452 (0.316)	< 0.001
SII	844.04 (531.4)	1564.20 (943.9)	< 0.001
CRP	17.50 (6.15)	85.5 (22.2)	< 0.001
Albumin	34.7 (31.7)	26.5 (21.8)	0.001

NLR, neutrophil-to-lymphocyte ratio; PLR, platelet-to-lymphocyte ratio; MLR, monocyte-to-lymphocyte ratio; SII, systemic immune inflammatory index; CRP, C-reactive protein. Median (IQR) values was compared using Mann-Whitney U test. A *p*-value < 0.05 was considered statistically significant.

## 4 Discussion

Pneumonia is considered community-acquired when it develops in the normal community setting without contact with the healthcare system. In contrast, community-onset healthcare-associated pneumonia is defined as pneumonia that develops in any patient who has been hospitalized for at least 2 days in the last 3 months, resides in a nursing home or long-term care facility, or has recently received antibacterial therapy, chemotherapy, dialysis, or wound care in the last 30 days. This differentiation is important because the spectrum of causal pathogens and thus the management and prognosis are different (9).

In our study, most patients presenting to the chest diseases outpatient clinic with a presumptive diagnosis of pneumonia were elderly, with nearly two-thirds being over 65 years of age. A substantial proportion of patients required hospitalization, including admission to the ward or intensive care unit, reflecting the severity of clinical presentations in our cohort. In addition, the majority of patients had at least one comorbidity, most commonly chronic lung disease and/or immunosuppressive conditions, as well as recent antibiotic exposure that may require hospitalization or healthcare contact. Accordingly, most patients in our cohort fulfilled the criteria for community-onset healthcare-associated pneumonia. This finding is likely related to the tertiary care setting of our institution, where patients with more complex clinical conditions and higher disease severity are preferentially referred. In contrast, patients with uncomplicated community-acquired pneumonia are more likely to be diagnosed and treated at primary or secondary healthcare levels. This referral pattern should be taken into account when interpreting the microbiological spectrum and clinical outcomes of our study.

Among the scoring systems used to assess disease severity in community-onset pneumonia and to guide decisions regarding hospitalization to the ward or intensive care unit, the PSI is one of the most widely applied tools. Previous studies evaluating comorbid conditions associated with CAP have shown that, in the absence of respiratory diseases or immunosuppressive conditions, pneumonia tends to be less severe and can often be managed with outpatient treatment. In contrast, chronic lung diseases–particularly chronic obstructive pulmonary disease (COPD) and asthma–are consistently reported as the most common comorbidities among hospitalized CAP patients, while the ranking of the second and third most common comorbid conditions varies between studies. Jain et al. (7) identified chronic lung disease (42%), chronic heart disease (35%), immunosuppression (30%), and diabetes mellitus (26%) as the most frequent underlying conditions in HCAP patients. Similarly, Alonso et al. (10) reported immunocompromised status, neurological disease, and chronic renal failure as the leading risk factors in this population. In another cohort study of HCAP patients, a high comorbidity index was found to be a stronger predictor of all-cause mortality than PSI, with 40% of patients having underlying lung diseases, including COPD and asthma (11). Consistent with previous reports, chronic lung disease and immunosuppressive status were highly prevalent in our cohort. Overall, 66.1% of patients had chronic lung disease and 37.9% had an immunosuppressive condition. Among hospitalized patients, these rates were 62.7% and 42.7%, respectively. Notably, PSI scores did not differ significantly according to the presence of chronic lung disease, immunosuppressive status, or their combination.

During the COVID-19 pandemic, several studies focused on bacterial pneumonia developing after SARS-CoV-2 infection and evaluated factors associated with disease severity and mortality. Maruschak et al. (12) reported a correlation between PSI and comorbidity burden in patients who were hospitalized with CAP following recent SARS-CoV-2 infection. In that study, the most common comorbidities were congestive heart failure, moderate-to-severe liver disease, and diabetes mellitus with chronic complications. In our study, no correlation was observed between comorbidity burden and PSI among patients with pneumonia following COVID-19; however, the small number of post–COVID-19 CAP cases represents a limitation of our study.

Pneumonia Severity Index which is widely used to assess disease severity in community-acquired pneumonia and to guide decisions regarding hospitalization, is an age-dependent scoring system. Therefore, a high PSI score in younger patients may represent an alarm situation (9). In our study, high PSI scores were observed in 37.8% of patients aged ≤65 years; all of these patients were between 45 and 65 years of age and had at least one pulmonary and/or extrapulmonary comorbidity. Notably, in this younger age group, immunosuppressive conditions were relatively frequent (58.8%), with malignancy being the predominant underlying condition (47.1%). In contrast, among patients older than 65 years with high PSI scores, immunosuppressive conditions were less frequent (36.1%) and were predominantly related to diabetes mellitus (72.7%). These findings suggest that the profile of immunosuppressive conditions associated with high PSI differs by age group. Accordingly, heightened clinical attention may be warranted not only for younger patients with malignancy but also for older patients with diabetes mellitus who present with high PSI scores.

Although *Streptococcus pneumoniae* is reported as the most common causative agent in classical community-acquired pneumonia (CAP) cohorts Gram-negative pathogens are more frequently encountered in healthcare-associated pneumonia (7, 13, 14). In our study, the predominance of Gram-negative bacteria, particularly *Pseudomonas aeruginosa*, likely reflects the high comorbidity burden and tertiary care setting of our institution. *P. aeruginosa* was the most frequently isolated pathogen in both outpatients and inpatients, and most affected patients had underlying chronic lung disease or immunosuppressive conditions, which are recognized risk factors for *P. aeruginosa* infection. Reported rates of *P. aeruginosa* pneumonia vary across studies, ranging from 4% to 5% in immunocompromised CAP patients to 17% in healthcare-associated pneumonia cohorts (15–20). These findings suggest that patients with uncomplicated CAP are largely managed at primary and secondary healthcare levels, whereas our tertiary care center predominantly follows older patients with multiple comorbidities, consistent with a community-onset healthcare-associated pneumonia population.

Antibiotic susceptibility results are essential for evaluating the adequacy of empirical therapy and determining the need for treatment modification. Current American Thoracic Society and Infectious Diseases Society of America (ATS/IDSA) Guidelines recommend a combination of a beta-lactam and a macrolide or a respiratory fluoroquinolone for empirical treatment of community-acquired pneumonia, however, in the presence of *Pseudomonas* risk factors, this regimen should be replaced with an antipseudomonal beta-lactam, such as cefepime or ceftazidime (21). In our study, no resistance was detected among common CAP pathogens such as *S. pneumoniae*, *H. influenzae*, and *M. catarrhalis* to cefotaxime and ceftriaxon (third-generation cephalosporins) frequently used in empirical therapy. In contrast, resistance rates of 30%–35% were observed to antipseudomonal cephalosporins, largely reflecting the predominance of *P. aeruginosa*, which is intrinsically resistant to cefotaxime and ceftriaxone. While no levofloxacin resistance was observed in pneumococci, high resistance rates were detected in *H. influenzae*, *Enterobacteriaceae*, and *P. aeruginosa*. These findings underscore the importance for healthcare institutions to consider their own local data, including pathogen spectrum and antimicrobial resistance surveillance, in conjunction with current guideline recommendations when determining empirical antibiotic therapy.

Even today, sputum cultures are still not requested enough from patients with pneumonia in most outpatient settings, and the effectiveness of empirical therapy may not be adequately assessed, potentially leading to treatment failure and usage of antibotics with larger effect spectrum. Therefore particularly in developing countries, multidrug resistance among infectious agents is increasing, largely due to frequent and inappropriate antibiotic use. Our country remains among those with the highest rates of antibiotic consumption worldwide (22), underscoring the importance of antimicrobial resistance surveillance for each bacterial pathogen. In our study, multidrug resistance was most frequently observed in *Klebsiella* spp. (77.4%) and *Escherichia coli* (66.7%), followed by *Haemophilus influenzae* (50%) and *Pseudomonas aeruginosa* (32.5%). In comparison, a methodologically similar study conducted by Kamori et al. (23) reported MDR pathogens in 47% of sputum samples, with lower MDR rates among Gram-negative bacteria.

Although studies examining the relationship between MDR and the PSI are limited, we found significantly higher mean PSI scores in patients with MDR pathogens compared to those without MDR. Patients with higher PSI scores are more likely to have prior healthcare exposure, frequent hospital admissions, and multiple comorbidities, which may predispose them to colonization or infection with multidrug-resistant pathogens. Therefore, the observed association between higher PSI scores and MDR pathogens may reflect confounding or reverse causation rather than a direct causal relationship.

Based on laboratory findings, inflammatory markers including the NLR, PLR, monocyte-to-lymphocyte ratio (MLR), systemic immune–inflammation index (SII), and C-reactive protein (CRP) were significantly higher in patients with higher PSI scores. Previous studies have demonstrated the prognostic value of these markers in pneumonia. In one study, NLR was found to be superior to PSI in predicting 30-days mortality, with no deaths observed among patients with an NLR below 11.12 (24). Similarly, a systematic review of 186 studies reported that an NLR value above 10 was associated with increased mortality and showed better predictive performance than CRP, neutrophil count, and PSI (25).

In a large retrospective study including 6,802 patients with pneumonia, SII was identified as a significant predictor of both the need for mechanical ventilation and mortality (26). Moreover, a comprehensive review reported that SII differed significantly in conditions such as malignancy and infection, supporting its role as a clinically relevant biomarker (27). Although mortality outcomes were not assessed in our study, the observed associations between higher PSI scores and elevated NLR, PLR, MLR, SII, and CRP levels are consistent with findings reported in the literature.

Our study contributes to the literature by providing 5-years data from the only center in the province capable of performing sputum cultures and by offering a detailed evaluation of the effects of various comorbid conditions (including diabetes mellitus, organ transplantation, malignancy, and autoimmune diseases), etiological agents, and multidrug resistance on PSI scores. Unlike many previous studies, our findings are based exclusively on culture-proven results, which strengthens their clinical relevance. However, the study has several limitations. First, the exclusion of numerous culture-positive results–particularly repeated isolations of the same pathogen in patients with underlying conditions such as COPD–resulted in a relatively small number of eligible culture-confirmed cases despite the 5-years study period. This may have reduced statistical power and limited generalizability. As all available culture-proven cases within the study period were included, no *a priori* power calculation was performed; thus, the sample size reflects the maximum obtainable population rather than a predefined estimate. Due to the retrospective design, selection bias cannot be entirely excluded, and causal relationships cannot be definitively established; the results should therefore be interpreted as associative rather than causal. The retrospective design precludes definitive causal inference and introduces the potential for selection bias; therefore, the findings should be interpreted as associative. The predominance of male patients may also influence interpretation of the findings, although this distribution likely reflects the patient population presenting to our hospital. Additionally, the low number of patients without underlying comorbidities precluded the inclusion of a true control group. As a single-center study in a tertiary healthcare setting, the findings may not be generalizable to other regions or to primary and secondary care populations, given the predominance of patients with multiple comorbidities and the absence of a control group. Multivariable analysis was not performed; therefore, the findings should be interpreted as descriptive associations rather than independent predictors. Another important limitation is the lack of cause-specific outcome and mortality data, which prevented analysis of short-term or 30-days pneumonia-related mortality. Finally, given the comorbidity burden and prior healthcare exposure of most patients, many cases fulfilled criteria for community-onset healthcare-associated pneumonia, which may influence pathogen distribution and treatment patterns and should be considered in the interpretation of the findings.

## 5 Conclusion

In this study, clinical, laboratory, and microbiological factors associated with PSI were evaluated in patients with culture-proven bacterial pneumonia. Multidrug-resistant pathogens, increased immune–inflammatory markers, hypoalbuminemia, and comorbidity burden were associated with higher PSI scores supporting their potential role in identifying patients at risk for more severe disease. These findings highlight the importance of considering community-onset healthcare-associated pneumonia and of incorporating local antimicrobial resistance surveillance into empirical treatment decisions. Further multicenter prospective studies are warranted to validate these associations and to clarify their prognostic significance.

## Data Availability

The raw data supporting the conclusions of this article will be made available by the authors, without undue reservation.
